# Curcumin Decreases Hippocampal Neurodegeneration and Nitro-Oxidative Damage to Plasma Proteins and Lipids Caused by Short-Term Exposure to Ozone

**DOI:** 10.3390/molecules26134075

**Published:** 2021-07-03

**Authors:** María Luisa Mendoza-Magaña, Hugo Alejandro Espinoza-Gutiérrez, Sendar Daniel Nery-Flores, Abraham Alberto Ramírez-Mendoza, Cesar Ricardo Cortez-Álvarez, Robert de Mario Bonnet-Lemus, Mario Alberto Ramírez-Herrera

**Affiliations:** 1 Laboratorio de Neurofisiología, Departamento de Fisiología, Centro Universitario de Ciencias de la Salud, Universidad de Guadalajara, Guadalajara, Jalisco ZC 44340, Mexico; mmendoza@cucs.udg.mx (M.L.M.-M.); hugo.espinoza2323@alumnos.udg.mx (H.A.E.-G.); abrahamalberto.ramirez@alumnos.udg.mx (A.A.R.-M.); robert.bonnet@alumnos.udg.mx (R.d.M.B.-L.); 2 Departamento de Investigación en Alimentos, Facultad de Ciencias Químicas, Universidad Autónoma de Coahuila, Saltillo, Coahuila ZC 25280, Mexico; sendar_nery@uadec.edu.mx; 3 Departamento de Farmacobiología, Centro Universitario de Ciencias Exactas e Ingenierías, Universidad de Guadalajara, Guadalajara, Jalisco ZC 44430, Mexico; cesar.cortez@academicos.udg.mx

**Keywords:** curcumin, neurodegeneration, hippocampus, ozone, nitro-oxidative damage

## Abstract

Neurodegeneration is the consequence of harmful events affecting the nervous system that lead to neuronal death. Toxic substances, including air pollutants, are capable of inducing neurodegeneration. Ozone (O_3_) is the most oxidative toxic pollutant. O_3_ reacts with cellular components and forms reactive oxygen and nitrogen species, triggering nitro-oxidative damage during short-term exposure. Curcumin (CUR) is a natural phenolic molecule bearing well-documented antioxidant and anti-inflammatory biological activities in diverse experimental models. The aim of this work was to evaluate the effect of preventive dietary administration of CUR against hippocampal neurodegeneration and nitro-oxidative damage caused by short-term exposure to O_3_. Eighty Wistar male rats were distributed into four experimental groups, twenty rats each: intact control; CUR dietary supplementation without O_3_ exposure; exposure to 0.7 ppm of O_3_; and exposed to O_3_ with CUR dietary supplementation. Five rats from each group were sacrificed at 1, 2, 4, and 8 h of exposure. The CUR dose was 5.6 mg/kg and adjusted according to food consumption. CUR significantly decreased oxidative damage to plasma lipids and proteins, as well as neurodegeneration in CA1 and CA3 hippocampal regions. Concluding, CUR proved effective protection in decreasing neurodegeneration in the hippocampus and prevented systemic oxidative damage.

## 1. Introduction

Neurodegeneration occurs in diseases such as Parkinson’s and Alzheimer’s as a consequence of complex pathophysiological processes involving oxidative damage [1,2]. Many toxic substances, including air pollutants, are capable of inducing neurodegeneration. Air pollution refers to the anomalous presence of toxic solid, liquid, and gaseous particles suspended in the troposphere [3]. Likewise, air pollution has an anthropogenic origin due to the consumption of fossil fuels and the generation of toxic residuals [4]. Among the harmful pollutants, ozone (O_3_) stands out for its oxidative capacity even at short exposure times (hours) [5,6,7].

The sustained overproduction of reactive oxygen and nitrogen species (RONS) caused by O_3_ exposure decreases the activity of the endogenous antioxidant systems, making the brain particularly susceptible [8,9,10]. This means that oxidative damage would be the first step that leads to irreversible modifications of the cellular structure and function, impairment of cell repairing processes, mitochondrial dysfunction, cell degeneration, and finally, death [11,12]. Specifically, in the central nervous system (CNS), oxidative damage induces metabolic alterations, altered neurogenesis, reactive astrocytosis, neurotransmitter imbalance, and failure in ATP production [13].

RONS can cause damage via two distribution routes: first, a direct route through the olfactory mucosa, olfactory bulb, entorhinal cortex, hippocampus, and then to the rest of the CNS. Oxidative damage directly affects the hippocampus, which is particularly susceptible, and thus important cognitive functions such as memory and learning could be impaired [14]. Simultaneously, the second route is through the pulmonary and systemic circulations, affecting the blood-brain barrier and disseminating the oxidative damage throughout the whole body [15]. This means that, in both cases, RONS indiscriminately oxidizes and nitrosylates proteins and produces lipid peroxidation (LPO), resulting in cell dysfunction and death [16,17].

In this scenario, neuroprotective and RONS scavenging activities are needed. They can be achieved by the consumption of exogenous antioxidant agents that directly neutralize RONS and enhance the activity of the endogenous antioxidant system [18]. Natural antioxidants could be used as preventive and therapeutic complements against various degenerative diseases.

Although polyphenols demonstrate antioxidant activity, a good candidate must meet specific requirements, such as electrophilic functional groups (susceptible to nucleophilic attacks), free electronic pairs, resonant forms, proton donor, an affinity for cellular ligands that increase the activity of endogenous antioxidants (indirect antioxidant activity), and metal chelation to avoid catalysis of oxidative reactions by metals [19]. Therefore, only certain molecules, such as neurodegeneration cover these requirements.

Curcumin (CUR) is a natural molecule, chemically defined as diferuloylmethane, which is isolated from the rhizome of *Curcuma longa*. CUR has demonstrated direct and indirect antioxidant activity because it acts as a scavenger for RONS, and in vivo, it enhances the activity of superoxide dismutase, catalase, gluthathione peroxidase, and hemeoxigenase-1. Furthermore, CUR is capable of interacting with growth factors, receptors, transcription factors, ion channels, cytokines, enzymes, and genes [20,21,22,23]. However, if ozone is capable of producing oxidative stress during short-term exposure, then it is necessary to evaluate if CUR has the ability to reduce the damage and its further consequences.

Therefore, the present study aimed to analyze the neuroprotective effect (neurodegeneration in rat hippocampus) and antioxidant effect (lipid peroxidation, protein oxidation, and protein nitrosylation in rat plasma) exerted by CUR on a short-term damage model caused by O_3_. These experiments emulate at least one of the possible conditions in an environmental contingency where the concentration of O_3_ was raised during a brief period of time, as this phenomenon has been reported to cause neurodegeneration through oxidative damage. Repeated episodes such as this could lead to the development and onset of neurodegenerative diseases in human beings living in polluted cities. Furthermore, the preventive dietary supplementation with CUR may offer neuroprotection that should be evaluated in clinical assays.

## 2. Results

### 2.1. Curcumin Reduces Neurodegeneration in Rat Hippocampus

#### 2.1.1. Silver Staining

Neurodegeneration was determined by quantification of positive cells that underwent structural loss and are detectable by silver staining in the CA1 and CA3 hippocampus regions.

**Figure 1:**
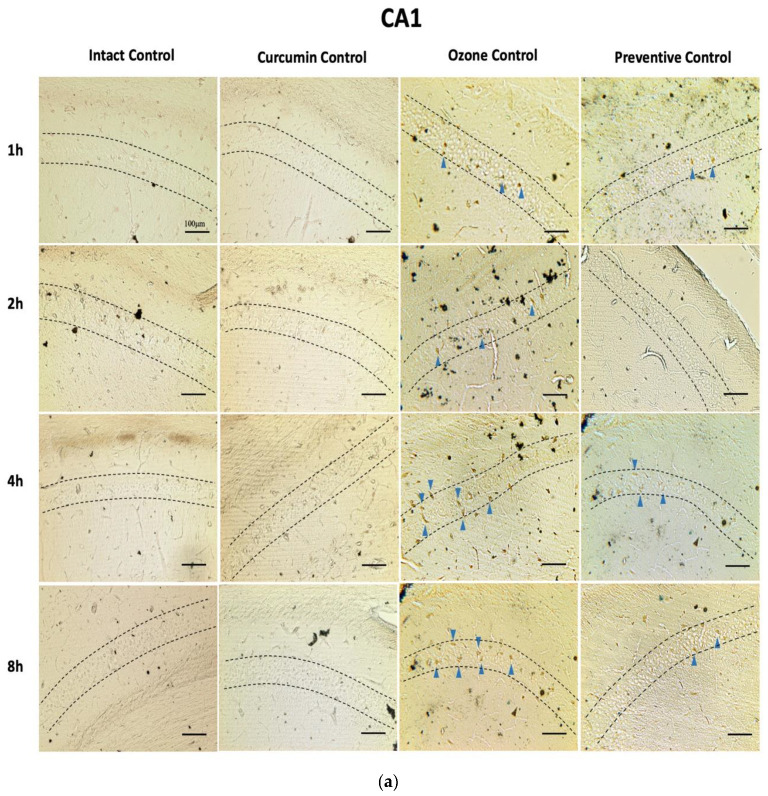

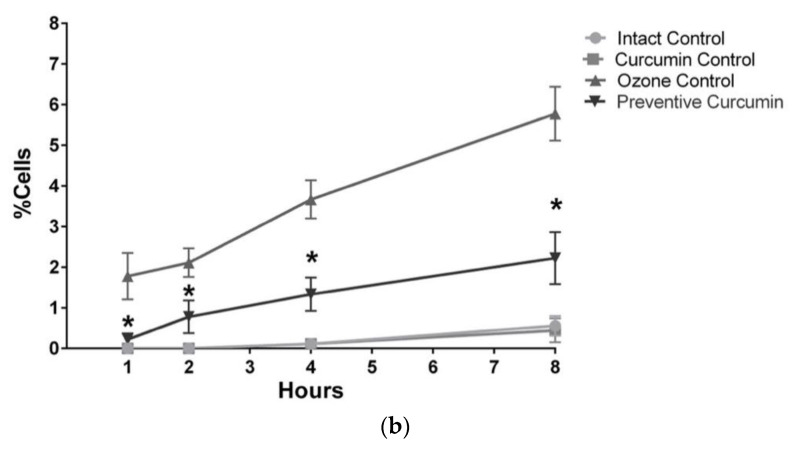
Effect of CUR against hippocampal neurodegeneration after short-term O_3_ exposure (Table S1). CA1 region was evaluated by a modified silver technique (de Olmos stain). (**a**) This panel shows representative images of tissue sections at different O_3_ exposure times in experimental groups (*n* = 5), showing positive cells (dark brown), indicated with blue arrowheads, dashed lines indicate the CA1 area. (**b**) The graph illustrates the comparison of the degenerative cell percentage at each exposure time in experimental groups; results are expressed as mean values ± SEM, and the asterisk always indicates a statistical difference between the preventive curcumin and the ozone control groups (*p* < 0.05). Data of intact control and curcumin control groups are overlapped in the two lines at the bottom.

In CA1 (Figure 1) after 1 h of exposure, the OC group began to present neurons in degeneration (1.778 ± 0.572%) this increase is significant compared to the IC (0.00 ± 0.00% *p* < 0.005) and CC groups (0.00 ± 0.00% *p* < 0.005). The PC treatment group with dietary curcumin supplementation and O_3_ exposure showed a significant decrease in the percentage of positive cells compared to the OC group (PC 1 h 0.222 ± 0.147% *p* < 0.05).

After 2 h of exposure, the OC group displayed a greater percentage of neuronal cells in degeneration (2.111 ± 0.351%) with respect to the control groups IC and CC (IC and CC 2 h 0.00 ± 0.00% *p* < 0.0005). The PC group presented a lower number of positive neurons than the OC group, meaning that the PC treatment group with dietary CUR supplementation significantly reduced neurodegeneration in CA1 after 2 h of exposure in respect to the OC group (PC 2 h 0.778 ± 0.401, *p* < 0.0005).

At 4 h of exposure in the CA1 region of the hippocampus, the OC group showed a higher neurodegenerative status caused by O_3_ exposure (3.667 ± 0.471%) compared to the control group IC (0.111 ± 0.111% *p* < 0.0001), meaning it had a greater number of cells in degeneration. The PC group that received treatment with CUR and O_3_ exposure displayed a significant decrease in the percentage of neuronal cells in degeneration with respect to the OC group (PC 1.333 ± 0.408% *p* < 0.005).

In the CA1 region of the hippocampus at 8 h of exposure, the OC and IC groups presented significant differences in the neurodegenerative status because the OC group had a greater percentage of positive cells (5.778 ± 0.662% *p* < 0.0001) with respect to the IC group (0.556 ± 0.242%) because of O_3_ exposure. In addition, the PC group that received CUR prior to exposure significantly reduced the number of degenerative cells (2.222 ± 0.641% *p* < 0.005) with respect to the OC group.

**Figure 2:**
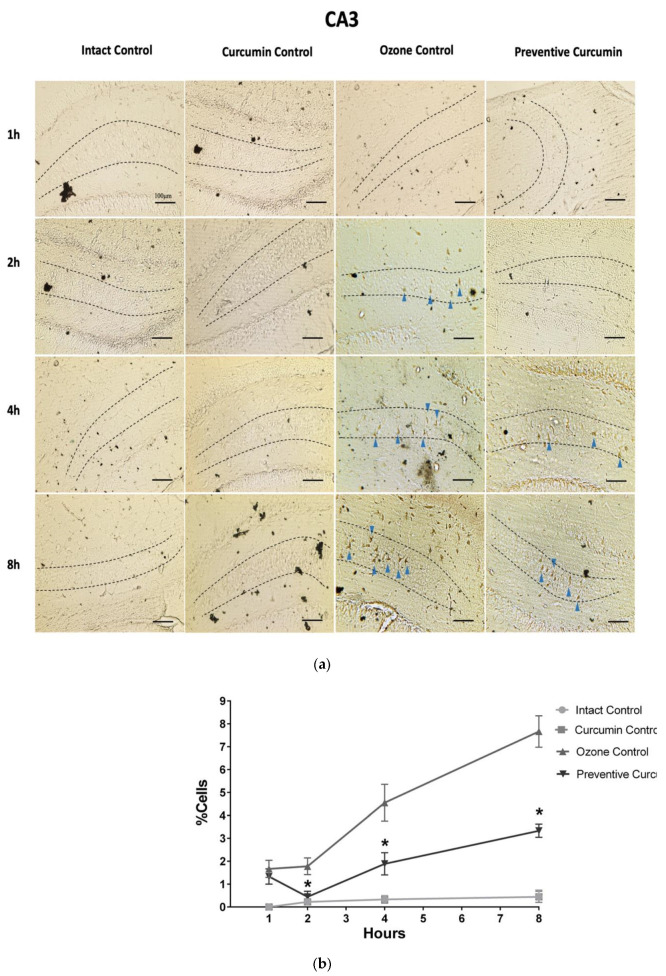
Effect of CUR against hippocampal neurodegeneration after short-term O_3_ exposure (Table S2). CA3 region was evaluated by a modified silver technique (de Olmos stain). (**a**) This panel shows representative images of tissue sections at different exposure times in experimental groups (*n* = 5) showing positive cells (dark brown), indicated with blue arrowheads, dashed lines indicate the CA3 area, (**b**) The graph illustrates the comparison of the degenerative cell percentage at each exposure time in experimental groups; results are expressed mean values ± SEM and the asterisk indicates a statistical difference between the preventive curcumin and ozone control groups (*p* < 0.05) at 2, 4, and 8 h. Data of intact control and curcumin control groups are overlapped in the two lines at the bottom.

In the CA3 region (Figure 2) after 1 h of exposure, the OC group presented more neuronal cells in degeneration (1.667 ± 0.373%) compared to the IC (0.00 ± 0.00%, *p* < 0.005) and CC groups (0.00 ± 0.00%, *p* < 0.005). The PC group that received a diet with CUR and O_3_ exposure showed a different pattern in contrast to the OC group. However, the number of positive neurons was not significantly reduced (1.333 ± 0.333%, *p* > 0.05).

At 2 h of O_3_ exposure in the CA3 region, a significant increase in degenerating neurons was observed in the OC group (1.778 ± 0.364%) compared to the IC (0.222 ± 0.147% *p* < 0.005), such as what was observed in CA1. In addition, the PC group showed the protective effect of CUR against the damage caused by O_3_ (0.444 ± 0.242%, *p* < 0.01), the PC treatment group showed a significant decrease of neurons in degeneration.

When animals were exposed to 4 h, the OC group showed more neuronal cells in degeneration (4.556 ± 0.801%) compared to the non-exposed IC group (0.333 ± 0.167% *p* < 0.0005). O_3_ significantly increased the number of affected cells. The PC group showed that CUR significantly reduced the number of cells in degeneration compared to the OC group (1.889 ± 0.484% *p* < 0.05).

In the CA3 region of the hippocampus at 8 h of O_3_ exposure, the OC group showed a significant increase in the number of cells in degeneration (7.667 ± 0.687%) versus the IC (0.444 ± 0.294% *p* < 0.0001). However, the PC group with a diet supplemented with CUR and O_3_ exposure showed a decrease in the number of degenerating neuronal cells (PC 3.333 ± 0.289% *p* < 0.0001) with respect to the OC group. The OC group showed an increase in the percentage of positive cells, and that CUR reduced that number.

The PC group showed that CUR decreased the number of neuronal cells in degeneration with respect to OC from 1 to 8 h of exposure (PC 1h 1.333 ± 0.333% *p* > 0.05; PC 2 h 0.444 ± 0.242% *p* < 0.01; PC 4 h 1.889 ± 0.484% *p* < 0.05 and PC 8 h 3.333 ± 0.289% *p* < 0.0001). Therefore, the diet supplemented with CUR exerted a neuroprotective effect against O_3_ exposure.

#### 2.1.2. Fluoro-Jade C Stain

Neuronal degeneration status was also determined by quantification of positive cells based on the FJC-polyamine binding that allows visualization of early degenerative changes in CA1 and CA3 hippocampus regions.

**Figure 3:**
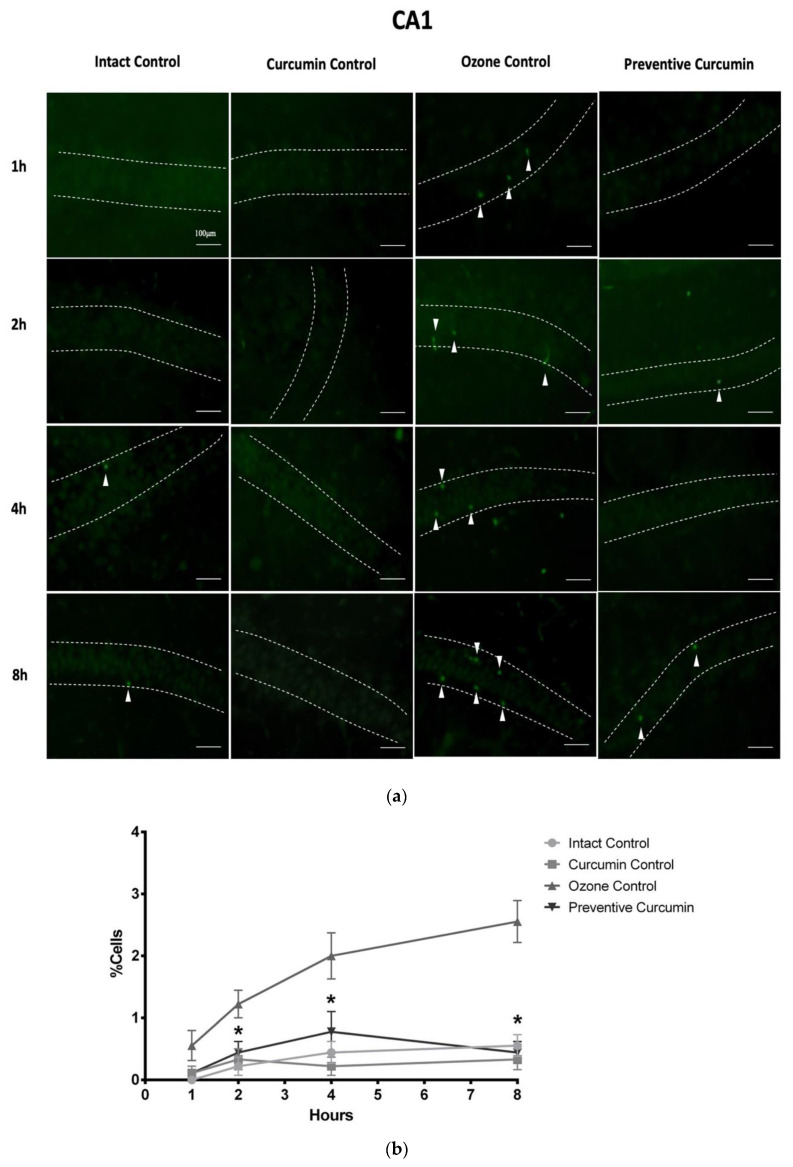
Effect of CUR on hippocampal neurodegeneration after short-term O3 exposure (Table S3). CA1 region was evaluated by the Fluoro-Jade C stain. (**a**) The panel shows representative images of brain sections at different exposure times in experimental groups (*n* = 5) showing positive cells (green fluorescence), indicated with white arrowheads, dashed lines indicate the CA1 area. (**b**) The comparison of the degenerative cell percentage at each exposure time in the experimental groups is shown; results are expressed as mean values ± SE, and the asterisk indicates a statistical difference between the preventive curcumin and ozone control groups (*p* < 0.05) at 2, 4, and 8 h.

In the CA1 region (Figure 3), after 1 h of exposure, the IC and CC groups did not present a notable degenerative process, while the OC group exposed to O_3_ showed cells in degeneration. The group that received CUR and exposure to O_3_ (PC) presented a pattern similar to that observed in the IC and CC groups. The OC group displayed a greater presence of positive cells to the FJC stain (0.556 ± 0.242%). However, this increase was not significant compared to the IC (0.000 ± 0.000), CC (0.111 ± 0.111%), and PC groups (0.111 ± 0.111%).

After 2 h of exposure in CA1, the IC group receiving O_3_-free air had baseline levels of the degenerative process, this same effect was observed for the CC group. The OC group that was exposed for 2 h to O_3_ showed a greater presence of positive cells compared to the previous groups. Finally, the PC group that was treated with CUR showed a lower number of cells positive to the FJC stain. In summary, there was a greater presence of degenerative cells in the OC group (1.222 ± 0.222%) compared to the IC (0.222 ± 0.147% *p* < 0.005) and CC groups. The PC group that received CUR in the diet prior to the O_3_ exposure phase showed a significant decrease in the percentage of cells positive to the degeneration process compared to the OC group (0.444 ± 0.176% *p* < 0.05).

At 4 h of exposure, both the IC and CC groups presented a background signal as faintly fluorescent neuronal cells. In contrast, the OC group observed a considerable increase in the number of positive cells to FJC. The diet with CUR prior to O_3_ exposure in the PC group displayed a clear reduction in the number of positive cells. Therefore, the percentage of cells in the process of degeneration after 4 h of exposure in the OC group showed a significant increase in FJC stain positive cells (2.000 ± 0.373% *p* < 0.01) compared to the IC (0.444 ± 0.176%). In addition, the OC group presented a significant decrease in neuronal degeneration with respect to the PC group (0.778 ± 0.324% *p* < 0.05).

After 8 h of exposure, the IC and the CC groups showed background levels of positive cells to the FJC dye. In the OC group exposed to O_3_, there was a notable increase in the presence of positive cells, which highlights the neurodegenerative process, while the PC demonstrated a clear reduction in the number of positive cells. When quantifying the number of cells in the degenerative process, the statistical analysis showed that the OC group presented a significant increase in the percentage of positive cells (2.556 ± 0.338% *p* < 0.001) in comparison with IC (0.556 ± 0.176%). The PC group showed a significant decrease in neuronal degeneration with respect to the OC group (0.444 ± 0.176% *p* < 0.001), which shows the neuroprotective effect of CUR against the toxic damage caused by O_3_.

**Figure 4:**
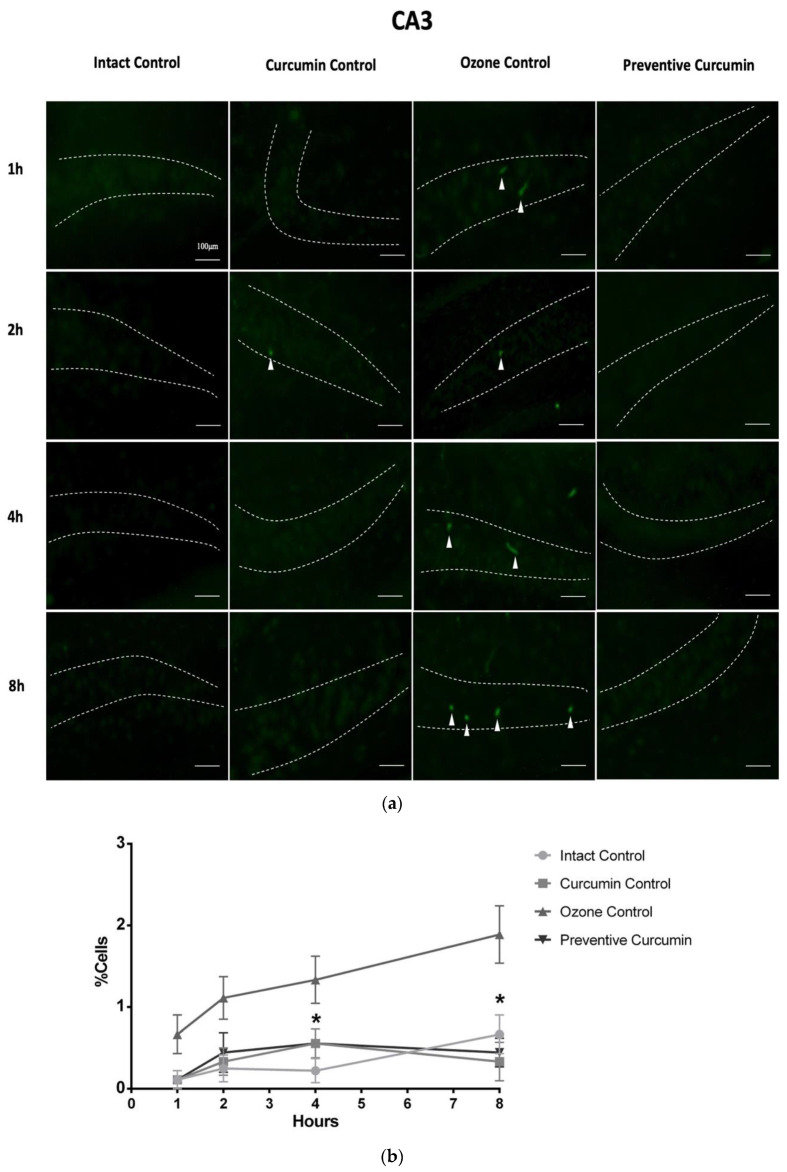
Effect of CUR on hippocampal neurodegeneration after short-term O_3_ exposure (Table S4). CA3 region was evaluated by the Fluoro-jade C stain. (**a**) The panel shows representative images of brain sections at different exposure times for each experimental group (*n* = 5) where positive cells (Green fluorescence) are indicated with white arrowheads, dashed lines indicate the CA3 area. (**b**) The graph illustrates the comparison of the degenerative cell percentage at each exposure time in the experimental groups; results are expressed as mean values ± SEM, the asterisk indicates a statistical difference between the preventive curcumin and ozone control groups (*p* < 0.05) at 4 and 8 h.

In the CA3 region (Figure 4), after 1 h of exposure to O_3_, the OC group showed a greater number of fluorescent cells (0.667 ± 0.236%) compared to the IC group (0.111 ± 0.111), however, without a significant difference (*p* > 0.05). The PC group decreased the percentage of fluorescent cells (0.111 ± 0.111) without a significant difference, compared to the OC group (*p* > 0.05), however a tendency to reduce the neurodegenerative status was observed. After 2 h of exposure, the OC group exhibited a large percentage of degenerated cells (1.111 ± 0.261% *p* < 0.05) compared to the IC group (0.222 ± 0.147%). However, the PC group (0.444 ± 0.242%) that received CUR prior to O_3_ exposure did not show a statistical difference with respect to the OC group but a tendency to decrease the degeneration process. After 4 h of exposure, O_3_ caused a significant increase of fluorescent cells in the OC group (1.333 ± 0.287, *p* < 0.01) compared to the IC group (0.222 ± 0.147), while the PC group showed a significant decrease in the percentage of neuronal cells in degeneration with respect to the OC group (0.556 ± 0.176% *p* < 0.05). After 8 h of exposure, the OC group showed a clear increase in the percentage of degenerative cells (1.89 ± 0.35% *p* < 0.05) compared to the IC group (0.67 ± 0.24). The group with the preventive administration of CUR (PC) decreased the percentage of fluorescent cells by the FJC stain (0.44 ± 0.18, *p* < 0.01).

### 2.2. Curcumin Reduced Lipid Peroxidation in Rat Plasma

**Figure 5:**
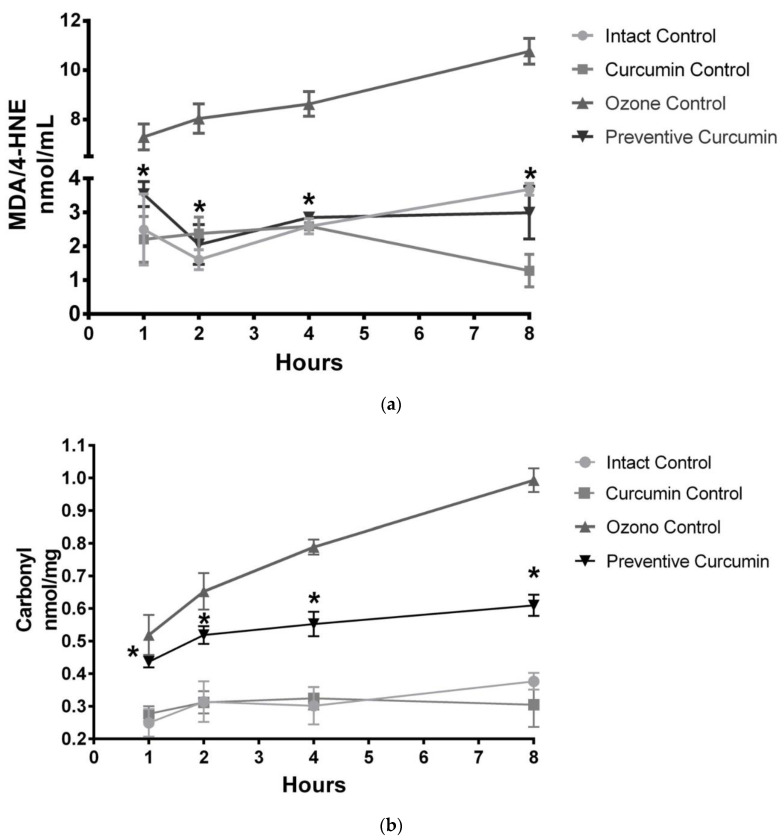
Antioxidant effect of CUR on lipid and protein damage by short-term exposure to O_3_ (Table S5A,B). (**a**) Plasma concentration of MDA/4-HNE (nmol/mL) for each experimental group (*n* = 5). (**b**) Plasma concentration of carbonylated proteins (nmol/mg) in the experimental groups (*n* = 5). Data represent the mean value ± SEM, and the asterisk indicates a statistical difference between the preventive curcumin and ozone control groups at all testing times for MDA/4-HNE concentration (*p* < 0.001) and protein carbonylation (*p* < 0.001).

The short-term exposure to O_3_ (OC group) showed a significant increase in the plasma concentration of MDA and 4-HNE (nmol/mL) (Figure 5a) from 1 to 8 h compared to the IC group. After 1 h of exposure to O_3_, the OC group displayed a significant increase of MDA and 4-HNE concentration (7.291 ± 0.526, *p* < 0.0001) compared to the IC group (2.491 ± 0.2704), while the PC group showed a significant decrease, compared to the OC group (3.537 ± 0.095% *p* < 0.0001).

A significant increase was also observed after 2 h of exposure to O_3_ (8.037 ± 0.596, *p* < 0.0001) compared to the IC group. The group with the preventive administration of CUR (PC) decreased the MDA and 4-HNE plasmatic concentration (2.050 ± 0.152, *p* < 0.0001). After 4 h, the OC group presented a significant increase in the plasmatic levels of MDA and 4-HNE (8.630 ± 0.498, *p* < 0.0001) compared to the IC group, while the PC group presented a significant decrease (2.849 ± 0.020, *p* < 0.0001) compared to the OC group. After 8 h of exposure to O_3_, the OC group showed a significant increase (10.7660 ± 0.522, *p* < 0.0001) with respect to the IC group (0.670 ± 0.240). This means that 1 h of exposure to 0.7 ppm of O_3_ is sufficient to produce oxidative damage to lipids that gradually increased for up to 8 h. The PC group showed a significant decrease after 8 h due to the antioxidant effect of CUR compared to the OC group (2.990 ± 0.200, *p* < 0.0001).

### 2.3. Curcumin Decreased Protein Oxidation in Rat Plasma

The short-term exposure to O_3_ showed a significant increase in carbonylated residues (nmol/mg) (Figure 5b) after 1 h of exposure as evidenced in the OC group (OC 1 h = 0.519 ± 0.016, *p* < 0.0001) with respect to the IC group (0.249 ± 0.011), while the PC group presented a significant decrease (0.519 ± 0.007, *p* < 0.0001). A significant increase was also observed after 2 h of exposure to O_3_ in the OC group (0.653 ± 0.014, *p* < 0.0001) compared to the IC group (0.314 ± 0.016). The group with the preventive administration of CUR (PC) significantly decreased the plasmatic concentration of carbonylated proteins (2.050 ± 0.152, *p* < 0.0001).

After 4 h, the OC group presented a significant increase (0.789 ± 0.006, *p* < 0.0001) compared to the IC group, while the PC group with preventive curcumin administration presented a significant decrease (0.538 ± 0.002, *p* < 0.0001). After 8 h of exposure to O_3_, the OC group showed a significant increase (0.993 ± 0.009, *p* < 0.0001) with respect to the IC group (0.377 ± 0.006) and the preventive administration of CUR against ozone exposure in the PC group displayed a significant decrease of carbonylated proteins (0.610 ± 0.008, *p* < 0.0001). In addition, both IC and CC did not show significant differences at all exposure times from 1 to 8 h.

### 2.4. Curcumin Decreased Nitrosylation of Rat Plasma Proteins

**Figure 6:**
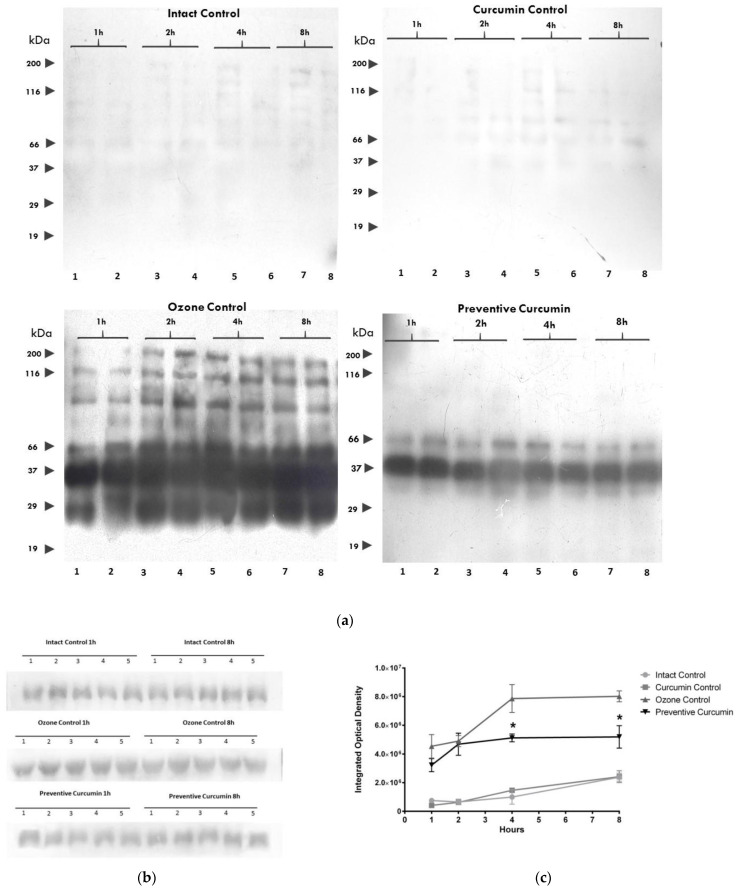
Effect of CUR against protein nitrosylation (Tirosin nitrosylation, 3-NT) caused by short-term ozone exposure (Table S6). (**a**) Detection of 3-NT in plasma proteins was performed by Western blot. A representative assay (*n* = 2) at different ozone exposure times is shown. In each assay, the labeled lanes correspond to the 3-NT profile from two different animals in the intact control, curcumin control, ozone control, and preventive curcumin groups. The arrows on the left side indicate the location of molecular markers. (**b**) Normalization for protein concentration in loaded samples for experimental groups was with an *n* value of 5 for each group at the exposure times. (**c**) The graph depicts the comparison of integrated optical density (arbitrary units) mean values and SEM obtained from 3-NT plasma protein bands at different ozone exposure times in the experimental groups (*n* = 5); the asterisk indicates a statistical difference between the preventive curcumin and ozone control groups (*p* < 0.05) at 4 and 8 h. The intact control and curcumin control groups showed low dispersion values and cannot be visualized at the used scale.

The densitometric analysis considered all bands across the entire lane. O_3_ caused a significant increase in immunoreactivity for 3-NT residues (Figure 6) from 1 to 8 has observed in the OC group compared to the IC group. After 1 h of exposure to O_3_ in the OC group, a greater IOD (4,536,000 ± 814,242, *p* < 0.0001) was observed compared to the IC group (756,800 ± 114,624), while the preventive administration of CUR in the PC group did not significantly decrease the immunoreactivity for 3-NT residues (3,228,000 ± 143,997, *p* > 0.05).

A significant increase was also observed after 2 h of exposure to O_3_ in the OC group (4,896,000 ± 392,619, *p* < 0.0001) compared to the IC group (653,000 ± 197,026). The group with the preventive administration of CUR (PC) did not significantly decrease the immunoreactivity for 3-NT residues (4,682,000 ± 243,195, *p* > 0.05).

After 4 h, the OC group presented a significant increase (7,866,000 ± 976,533, *p* < 0.0001) compared to the IC group, while the PC group with preventive curcumin administration presented a significant decrease (5,118,000 ± 86,059, *p* < 0.0001). After 8 h of exposure to O_3_, the OC group showed a significant increase (8,024,000 ± 146,500, *p* < 0.0001) with respect to the IC group (2,382,000 ± 282,492) and a significant decrease in nitrosative damage was found in the PC group with a diet supplemented with CUR prior to O_3_ exposure (5,188,000 ± 247,147, *p* < 0.0001). The CC and IC groups showed a similar behavior at all exposure times; therefore, the diet supplemented with CUR did not increase the nitrosative damage to proteins.

## 3. Discussion

The neuroprotective effects of CUR in a preventive approach against the damage caused by short-term exposure to O_3_ had never been reported emulating an event that may occur during a period of environmental contingency with high tropospheric O_3_ levels far above the maximum level allowed by the WHO [24]. Thus we exposed rats to a high concentration of O_3_ (0.7 ppm), which is reported as the annual mean exposure level in Mexico City [25]. The present study demonstrates the remarkable neuroprotective and antioxidant effects of CUR against hippocampal neurodegeneration.

In the model that we used, the route for entry of O_3_ is through nasal, olfactory, and alveolar mucosae, and at 0.7 ppm, the endogenous antioxidant system is rapidly overcome [26], leading to extensive oxidative damage. These processes initiate early neurodegenerative changes, as evidenced by the results obtained applying the modified de Olmos and the Fluoro-Jade staining techniques. Early neurodegenerative changes found after short-term O3 exposure are important, since, particularly in humans, neurodegeneration may lead to clinical manifestations in cognition, as during environmental exposure to O3 [27].

The short-term exposure to 0.7 ppm O_3_ increased the number of neuronal cells undergoing degeneration since the first hour of exposure. Likewise, it was observed that the most severe neuronal damage occurred after 8 h of exposure in both hippocampal regions. An apparent higher sensibility was found in the CA3 than in the CA1 region; however, this difference was not statistically significant. The fact that the silver stain and FJC yield similar results are concordant with those reported by Ye, et al. [28], who proved that the number of degenerating neurons and astrocytes increased in the hippocampus and temporal cortex in a model of transmissible spongiform encephalopathy in hamsters.

The Fluoro-Jade C staining is a technique based on the binding of this dye to polyamines allowing the detection of early degenerative changes. Meanwhile, the de Olmos silver staining method allows the detection of amorphous anionic debris in degenerating neuronal cells in the late stages of neurodegeneration [29]. These techniques are well accepted to detect degenerative changes. In general, we found that the FJC and the silver stain showed similar time-related results, but there were slight differences, mainly in the final number of degenerative cells detected. This suggests that O_3_ is capable of producing neurodegeneration in the hippocampus even at short exposure times.

In short-term O_3_ exposure, a main neurodegenerative process would involve the IL-1 receptor and the Toll 4 receptor (TLR4) activation by RONS [30], leading to NF-κB activation mainly in microglial cells. Furthermore, RONS cause apoptosis by the intrinsic pathway or by the caspase-12 activation in the presence of misfolded proteins in the rough endoplasmic reticulum [31,32]. Both processes are important (protein misfolding and neuronal apoptosis) characteristics of neurodegenerative diseases such as Alzheimer’s [33,34,35].

In the present study we found that CUR exerted neuroprotective activity, decreasing cellular degeneration. Our results match with previous studies of short-term neuronal damage triggered by an ischaemia/reperfusion in rats. A single preventive oral dose of 25 and 50 mg/kg of CUR was administered 1 h before occlusion of the left carotid artery for 30 min. Both doses decreased the number of immunoreactive cells to the pro-inflammatory enzyme COX-2 and pro-inflammatory interleukin TNF-α in the hippocampal regions CA1, CA3, and dentate gyrus. Consequently, the number of neurons in degeneration also decreased [21]. The previous results agree with the findings from our investigation, given that the preventive administration of CUR at a daily dose of approximately 5.6 mg/kg for 7 days decreased the number of degenerating hippocampal neurons after short-term exposure to 0.7 ppm O_3_ from 1 to 8 h. Thus, when acute damage occurs, a preventive approach has been found to have better results than the therapeutic approach [36].

The possible mechanisms by which CUR preventively acts in this model of acute damage by short-term O_3_ exposure are at first as a free radical scavenger molecule. Additionally, CUR reduces the expression of pro-inflammatory cytokines and apoptosis by downregulation of transcription factors, such as NF-kB [37], and upregulating the activation of the nuclear erythroid factor 2 (Nrf2), leading to the expression of genes involved in the antioxidant response and cellular detoxification of RONS [38,39]. These various mechanisms translate into the overall reduction of cell death and the decrease of detectable cell debris by the silver and FJC staining techniques.

The exposure to O_3_ caused increased LPO in a time-dependent manner starting at 1 h of exposure. This is consistent with other investigations where exposure to 1 ppm O_3_ for 4 h caused LPO in rat hippocampus [25]. Furthermore, Chen et al. [40] proved that in healthy humans, after 4 h of exposure to 0.2 ppm O_3_, the plasma concentration of the oxidant metabolite 8-isoprostane increased. Therefore, it has been demonstrated that O_3_ at concentrations from 0.2 to 1 ppm for 4 h is enough to increase LPO in rodents and humans.

In our results, the neutralization of RONS by CUR was evident since the first hour, additionally, at 2 h, the LPO apparently decreased to a lower level, and this could be attributable to early induction of antioxidant endogenous response by CUR. At 4 and 8 h, the profile was stabilized. Meanwhile, in the O_3_ control group, the LPO continued increasing. Previous studies have shown that CUR is able to decrease LPO in acute toxicity models by xenobiotics, such as parathion and paraquat derived from in vivo and in vitro studies, respectively. CUR was orally administrated at a dose of 200 mg/kg of body weight during the exposure to parathion. Meanwhile, in the in vitro study, SH-SY5Y cells were pretreated with 5–20 μM of CUR and exposed to 0.5 mM of paraquat [20,41]. This coincides with the results reported by Nery-Flores et al. [37], who proved that the preventive oral administration of CUR at a dose of 5.6 mg/kg/day reduced LPO in rat hippocampus after the acute O_3_ exposure (0.7 ppm/4 h for 15 days) and chronic O_3_ exposure (0.7 ppm/ 4 h for 60 days). These findings match our results since the preventive administration of a diet supplemented with CUR decreased the LPO in rats exposed to O_3_.

For the present investigation, we used an ELISA-based quantitative method to measure the extent of the damage to protein caused by short-term exposure to O_3_ in vivo, and we found that it increased as the time passed. Cross et al. [42] proved that short-term ex vivo exposure to O_3_ at 16 ppm for 2, 4, and 6 h increased the concentration of carbonylated residues in human plasma. The maximum carbonylation rate found in this study was at 6 h, while the maximum average concentration in our study was at 8 h. The divergence could be due to the exposure mode and the O_3_ concentration, but in both cases, a time-dependent effect occurs. Furthermore, intrinsic factors as age increase the susceptibility to oxidative damage. Prasada et al. [43] found that exposure to 0.25 and 1 ppm for 6 h a day, 2 days a week during 13 weeks in young rats (4 months old) and senescent rats (24 months old) showed protein carbonylation in the frontal cortex at both O_3_ concentrations, which were higher in the older animals.

The preventive administration of a diet supplemented with CUR exerted an important antioxidant effect on proteins by decreasing the plasma concentration of carbonylated amino acid residues during the short-term exposure to O_3_. These results match with those reported by Nery-Flores et al. [37], who proved that a preventive dietary administration of CUR reduced protein oxidation in rat hippocampus in acute O_3_ exposure (0.7 ppm/4 h for 15 days) and chronic O_3_ exposure (60 days). In the same study, CUR downregulated NF-κB.

The short-term oxidizing effect on proteins has also been studied by Borra, et al. [44]. Plasma samples from 50 male volunteers were incubated with Fenton reagent, and protein carbonylation was increased in comparison to the control group. These results match our findings because O_3_ exposure increased the concentration of oxidized proteins starting at 1 h. Borra et al. [44] observed that the protein oxidation decreased when CUR (50–200 µM) was added to plasma samples. Similarly, as it occurred in our study, the administration of CUR reduced oxidative damage to proteins starting at 1 h of exposure to O_3_.

Protein nitrosylation affects tyrosine residues by the peroxynitrite ion, forming 3-NT residues [45]. The presence of 3-NT alters protein structure and function, which suggests that this phenomenon occurs along with the oxidation of proteins [29,46,47].

In the present study, the exposure to O_3_ increased the detection of 3-NT residues starting at the first exposure hour. The highest nitrosative damage was reached at 4 h of exposure and did not increase after 8 h. This could be due to the presence of a limited number of tyrosine residues susceptible to nitrosylation. This phenomenon coincided with the observation by Kumarathasan, et al. [48], as the level of 3-NT did not increase when comparing the effect from exposure to 0.4 ppm or 0.8 ppm O_3_ after 4 h. Pinto-Almazán, et al. [49] reported that the exposure to 0.25 ppm O_3_ for 7 days and 4 h daily increased 1.5 times the concentration of 3-NT in rat hippocampus, while the exposure for 60 days was 2.3 times higher. Possibly at low O_3_ doses, oxidative processes are favored over nitrosative ones.

As already observed, we found that nitrosylation of protein was diminished by the dietary preventive administration of CUR. Braidy, et al. [50] induced glutamatergic excitotoxicity by quinolinic acid, and the administration of CUR reduced nitrosative damage in the quantification of positive cells and the intensity of 3-NT immunoreactivity. Moreover, a similar acute damage model with kainic acid that was treated with 50 mg/kg of CUR during 24 h in a preventive approach CUR has been shown to be able to prevent the increase of nitric oxide levels [46]. These results agree and support the evidence found in our study, where the preventive administration of CUR for 7 days, in vivo, decreased the concentration of 3-NT from the first hour of exposure to O_3_. However, this reduction was significant at 4 and 8 h of exposure. In a preventive approach, a possible mechanism of CUR is by the negative regulation of the transcription factor NF-κB [37] since the activation of NF-κB increases the transcription of various enzymes, such as inducible nitric oxide synthase, which in the presence of superoxide ion at physiological pH produces peroxynitrite ion (O_2_^−^ + NO → ONOO^−^) [47]. Another mechanism can be attributed to the radical scavenging action of CUR.

## 4. Materials and Methods

### 4.1. Animals

Animals were housed and handled in accordance with the guidelines and requirements of the World Medical Association Declaration of Helsinki and those established by the Ethical Committee of the Health Sciences Center at the University of Guadalajara in agreement with the NIH guidelines for the use of laboratory animals for experimentation (NIH Publications No. 8023, revised 1978). All experimental and analytical procedures were performed according to established guidelines. Eighty male Wistar rats (*Rattus norvegicus*), 21 days old and weighing ≈130 g, were used. We decided to evaluate the effects at this age because it has been reported that in older animals, there is oxidative damage inherent to aging, in such a way that the use of young animals allows mitigating the variation given by aging. Furthermore, young animals possess a robust endogenous antioxidant system, allowing us to prove that O_3_ at the exposure concentration used was strong enough to cause important damage at CNS and the systemic level [2]. The animals were kept in light/dark cycles 12 × 12 h, 22 ± 2 °C, and 50–60% relative humidity with free access to water and food (Prolab^®^RMH Laboratory Animal Diet, 2500 Rodent 5P14. St. Louis, MO, USA).

### 4.2. Diet

An alcoholic extract was obtained from commercial turmeric (Curcuma Kosher, Lot 09076, AMBE Phytoextracts PVT LTD, Delhi, India). The concentration of CUR in the extract was determined by UV spectrophotometry at 230 nm, and the molecular identity was determined by the infrared spectrum compared to a CUR standard (analytical grade, Sigma Chemical Co., St. Louis, MO, USA). The food pellets were impregnated with the alcoholic extract, and then the alcohol was eliminated by evaporation at 60 °C for 4 h in darkness. The homogeneous distribution of CUR in the food pellets was corroborated by UV-spectrophotometry. The pellets containing 200 ppm of CUR (0.16 mg/g food) were dispensed as described by Nery-Flores, Mendoza-Magaña, Ramírez-Herrera, Ramírez-Vázquez, Romero-Prado, Cortez-Álvarez and Ramírez-Mendoza [37]. Consequently, each rat received approximately a dose of 5.6 mg/kg/day.

### 4.3. Experimental Design

Animals were randomly distributed into four groups of 20 rats each. They were subjected to an adaptation period of seven days prior to the experiment to minimize the effect of human contact and lodging place in the experimental model. The intact control group (IC) was exposed to O_3_–free air without CUR supplementation; the CUR control group (CC) received food supplemented with CUR for 7 days during the adaptation period and was also exposed to O_3_-free air; immediately after the adaptation period, the O_3_ exposed control group (OC) received exposure to 0.7 ppm of O_3_ without CUR supplementation; and the preventive CUR group (PC) received food supplemented with CUR for 7 days during the adaptation period followed by exposure to O_3_. Five rats from each group were sacrificed at four exposure times (1, 2, 4, and 8 h).

Data of the IC group at the exposure periods represent the basal condition of the neurodegenerative state and oxidative marker profiles without O_3_ exposure nor supplementation with CUR. The results of the control groups, CC, represent the basal condition without exposure to O_3_ and fed with CUR supplementation. These groups were used to establish differences with the group exposed to O_3_ (OC) without CUR supplementation. Furthermore, they were used to compare the neuroprotective effect of CUR in the preventive approach with the group exposed to O_3_ and CUR supplementation (PC).

### 4.4. Ozone Exposure

Animals were exposed only once to O_3_ according to the established time (1, 2, 4, or 8 h) at a constant concentration of 0.7 ppm. They were placed in an acrylic hermetic chamber (65 × 25 × 45 cm L/H/D) connected to a gas premix chamber (40 × 24 × 45 cm) that received the O_3_ generated by a Certizon C100 apparatus (Sander, Elektroapparatebau GmbH, Uetze, Germany), connected to a source of medical grade oxygen. The O_3_ was then mixed with O_3_ free air to adjust to the aforementioned concentration. The O_3_ concentration was monitored with a semiconductor sensor (ES-600, Ozone Solutions Inc., Hull, IA, USA) to adjust the flow of oxygen and air needed for a proper atmosphere with a constant flow of 1.6–1.2 L/min. As part of a biosecurity safety process, the O_3_ expelled from the chamber was inactivated by passing it through a filter.

### 4.5. Tissue and Plasma Samples

Animals were anesthetized with a sodium pentobarbital intraperitoneal injection (36 mg/kg) after exposure. At this point, two simultaneous procedures were performed in the same animal: blood extraction and intracardial perfusion for tissue fixation.

Blood was extracted by intracardial punction with heparinized syringes and plasma was separated by centrifugation (3500 RPM/4 °C/10 min). Antiproteases (ethylenediamine tetra-acetic acid, ethylene glycol-bis(2-aminoethylether)-*N*,*N*,*N*′,*N*′-tetra-acetic acid, leupeptin, aprotinin, bestatin, and phenylmethanesulfonyl fluoride), mercaptoethanol, and butylated hydroxytoluene (Sigma Chemical Co., St. Louis, MO, USA), were added to samples and stored at −80 °C until analysis. The oxidative effect of inhaled O_3_ in short-term exposure could be detected easily and early in plasma components due to pulmonary function, and similar changes in CNS could be delayed to days [7,40].

After blood extraction, animals were intracardially perfused with 4% paraformaldehyde in PBS, and the brain was extracted. The whole brain was post-fixed in the same fixing solution for 48 h at 4 °C; then, it was placed in a cryoprotective solution and stored at 4 °C. Subsequently, 30 μm hippocampal coronal sections were cut using the Leica VT1000S vibratome between the stereotaxic coordinates of −6.04 mm to −2.30 mm with respect to Bregma. Tissue sections were stored with PBS 0.01 M and 0.0125% sodium azide at 4 °C until processed.

### 4.6. Neurodegeneration Silver Stain

Neurodegeneration occurs as a consequence of oxidative modification to biomolecules, leading to the generation of amorphous disintegrative debris detectable with silver stains [29,51]. Coronal brain sections (30 µm thick) were processed in flotation. After washing with _DI_ H_2_O, sections were immersed in the pre-impregnation solution (0.09% AgNO_3_, 0.65% allantoin, 0.93% dimethyl sulfoxide, 0.93% triethanolamine, 1.85% isopropanol, 0.04% CuNO3 in DI H2O) for 1 h at 37 °C. Afterward, sections were immersed in acetone and washed with DI H2O for 30 sec. The impregnation solution (3.25% AgNO3, 0.13% NaOH, 5.5% NH4OH, 0.4% acetone, 32% ethanol in DI H2O) was added at room temperature (RT) with agitation for 45 min. Subsequently, the developing solution was added (0.12% formalin, 0.007% citric acid, 10% ethanol in DI H2O) for 25 min at 37 °C with agitation. Sections were then washed with DI H2O for 5 min and 0.5% acetic acid for 5 min, three times in an alternate manner. Sections were left in DI H2O overnight at 4 °C to stabilize the silver aggregates. Next, sections were left in bleaching solution A (6% potassium ferricyanide, 6% potassium bromide and acidified with 250 µL 0.1N HCl) for 5 min with constant agitation at RT and washed with 0.05% Tris buffer solution-Tween (TBST) for 5 min and DI H2O for 5 min, three times, respectively. Then, sections were placed in bleaching solution B (0.06% potassium permanganate and 0.17% H2SO4 in DI H2O) for 20 sec, with agitation at RT and washed with 0.05% TBST and DI H2O for 5 min, 3 times in an alternated manner. Sections were placed in the stabilization solution (2% sodium thiosulfate) for 5 min with agitation at RT and washed 3 times with DI H2O for 5 min each at RT with agitation. The fixation solution (Kodak GBX fixer/replenisher, 1:6, Rochester, NY, USA) was added for 1 min at RT with agitation. Sections were washed three times with DI H2O for 5 min with agitation at RT. Brain sections were transferred to gelatin-coated glass slides and dried for 3 min in an oven at 70 °C. Sections were covered with buffered glycerin (50% glycerol, 50% 15.3 mM Na2HPO4) and mounted with coverslips. Digital images were taken with a CoolSnap-Pro Color video camera (Roper Scientific, Tucson, AR, USA) coupled to an inverted Olympus IX-71 microscope using the Image Pro Plus software version 6.0 (MediaCybernetics, Rockville, MD, USA). Finally, positive cells (dark brown) were quantified under the same conditions by two evaluators in the CA1 and CA3 regions of the hippocampus, counting the number of positive cells from a total of one hundred cells in three different fields per region with amplification of 200×. The fields were randomly selected.

### 4.7. Neurodegeneration Fluoro-Jade C Stain

Coronal brain sections (30 μm thick) were mounted on gelatin-coated glass slides and allowed to dry overnight at RT. They were immersed in a basic alcohol solution (20 mL of 5% NaOH in 80 mL in absolute ethanol) for 5 min and placed in 70% ethanol followed by _DI_ H_2_O for 2 min. Then, they were incubated with 0.06% potassium permanganate (KMnO_4_) solution for 20 min under gentle agitation. After washing for 2 min, samples were incubated with 0.0006% Fluoro-Jade C solution (AG325, Millipore, Burlington, MS, USA) in 0.1% acetic acid for 24 h. Sections were washed 3 times with _DI_ H_2_O and dehydrated in 70%, 90%, and 100% ethanol solutions, rinsed with xylene and mounted with synthetic resin for observation. Digital images were acquired using a CoolSnap-Pro Color video camera (Roper Scientific) coupled to an Olympus IX-71 inverted microscope using the Image Pro Plus software version 6.0 (MediaCybernetics). The percentage of degenerative cells was obtained by counting the number of positive cells from a total of one hundred cells in three different fields with an amplification of 200×. The count was carried out by two evaluators under the same conditions in regions CA1 and CA3. The fields were randomly selected.

### 4.8. Determination of Lipid Peroxidation

LPO assay determined the concentration of malondialdehyde (MDA) and 4-hidroxialkenal (4-HNE) according to the manufacturer’s instructions (Oxford Biomedical Res., Oxford, MI, USA). Briefly, plasma samples were centrifuged at 3000× *g* at 4 °C for 5 min, and 250 μL of each sample were transferred to assay tubes. They were mixed with 812.5 μL of *N*-methyl-2-phenylindole. Next, 187.5 μL of methanesulfonic acid was added, and samples were chilled in an ice bath and incubated at 40 °C for 45 min. Then, the reaction was stopped by chilling in an ice bath once again, and tubes were centrifuged at 15,000× *g* at 4 °C for 15 min. Samples were kept on ice, and 200 µL of supernatants were transferred in triplicate to a 96 well microplate, and absorbance was determined at 595 nm (EZ Read 400, Biochrom, Miami, FL, USA). The standard curve was prepared by adding 650 µL chromogen solution to increasing concentrations of 1,1,3,3-tetrametoxipropane (0.315–10 nmol/mL).

### 4.9. Determination of Oxidized Proteins

The plasma protein concentration was determined using the micro-Bradford method (Bio-Rad, Hercules, CA, USA), and absorbance was measured at λ 595 nm with a microplate reader (EZ Read 400, Biochrom, Miami, FL, USA).

This assay quantified the concentration of carbonyl groups in oxidized proteins, which reflects the oxidative damage caused by O_3_. Samples were thawed on ice and centrifuged at 3000 RPM for 5 min at 4 °C. Subsequently, plasma samples containing 100 μg of protein were mixed with 45 μL of 10 mM 2,4-dinitrophenylhydrazine (DNPH) and reacted in denaturing conditions (6M guanidine hydrochloride) at RT in the dark for 30 min with agitation. Afterward, 7.5 µL of stop solution (2M Tris and 30% glycerol) was added. Then, 5 µL of the previous reaction was mixed with 1 mL of carbonate-bicarbonate coating buffer, and 200 μL were loaded by triplicate in ELISA microplate wells and incubated at 4 °C overnight. Supernatants were discarded, and microplate wells were washed with phosphate buffer solution-Tween-20 (PBS-Tween) (0.05%). Next, samples were blocked with 0.1% reduced bovine serum albumin (rBSA) (0.1 g BSA, 2 g sodium borohydride in 100 mL _DI_ H_2_O) for two hours at 37 °C. After washing, rabbit anti-DNP (1:150, Millipore, 90,451) was added and incubated at 37 °C for 2 h. After washing, HRP labeled goat anti-rabbit (1:300, Li-cor, 926-80011) was incubated for 1 h at 37 °C. Wells were washed, and 200 µL/well of substrate reaction mix (bicarbonate-citrate buffer, 0.1% *o*-phenylenediamine, and 0.03% H_2_O_2_) was added and reacted at RT for 25 min with agitation in the dark. The reaction was stopped with 100 µL of H_2_SO_4_ 2.5M, then read at 490 nm (EZ Read 400, Biochrom, Miami, FL, USA). The standard curve was performed with oxidized BSA (oBSA) (1% BSA in 0.17 mM FeCl_3_, 22 mM sodium ascorbate) and 25 mM HEPES (4-(2-Hydroxyethyl) piperazine-1-ethanesulfonic acid, *N*-(2-Hydroxyethyl) piperazine-*N*′-(2-ethanesulfonic acid)), with a concentration range from 0 to 200 μg/μL (0 to 3 mM).

### 4.10. Detection of Nitrosylated Proteins

This assay was performed to detect the 3-nitrotyrosine (3-NT) residues in proteins [12,52]. Sample volumes of plasma containing 10 μg of protein mixed with Laemmli loading buffer were placed on 12% denaturing polyacrylamide gels and separated at 100 V. Next, proteins were transferred to a PVDF membrane at 0.35 A/4 °C for 105 min and then blocked with nonfat dry milk (Bio-Rad Blotting-Grade Blocker) for 2 h at RT. PVDF membranes were washed with 0.1% PBS-Tween and incubated overnight with rabbit anti-3-NT (1:2000, Invitrogen A21285, Carlsbad, CA, USA) at 4 °C. After 5 washes, membranes were incubated with HRP labeled goat anti-rabbit (1:25,000. Li-cor, 926-80011, Lincoln, NE, USA) for 1.5 h. After exhaustive washes, nitrosylated proteins were revealed by chemiluminescence, and a radiographic film was exposed, all experiments were revealed with the same exposure times (20 s), with luminol and hydrogen peroxide 1:1 (Immobilion Western, Millipore, WBKLS0500, Burlington, MS, USA), and with the same radiographic film (high-performance chemiluminescence, GE Healthcare, Chicago, IL, USA).To normalize the protein concentration in loaded samples, plasma specimens containing 10 μg of protein were electrophoresed in the same condition used for the 3-NT assay and then transferred to PVDF membranes and stained with Ponceau S red solution (0.1%).

The film was photographically revealed, and digital images were obtained and analyzed with the software Image Studio Lite Ver 5.2 (Li-cor, Lincoln, NE, USA) to determine the mean individual value of integrated optical density (IOD) per sample in the entire lane and results for each group at a given time were used to calculate the mean value for each group. Data were processed for statistical analysis. An acute neurotoxicity ozone exposure model exposed to 10 ppm for 3 days was used as a positive control [53].

### 4.11. Statistic Analysis

The data was processed using the GraphPad Prism (San Diego, CA, USA) statistical program version 6.01. Normality was checked using the Shapiro–Wilk normality test. Values were not normally distributed; thus, the Kruskall–Wallis non-parametric test was performed, followed by the Mann–Whitney U test. The results were expressed in means ± standard error of the mean (SEM), and a *p*-value of <0.05 was considered statistically significant.

## 5. Conclusions

The preventive dietary administration of CUR as an antioxidant and neuroprotective agent was effective in our experimental short-term oxidative model. These findings may be useful for the proposal of research projects in human populations living in densely polluted cities.

## Data Availability

Data supporting reported results can be found in the Research Coordination of the Centro Universitario de Ciencias de la Salud and with the Corresponding Author.

## Supplementary Materials

The following are available online, Table S1: Neurodegenerative changes in CA1 region by silver stain, Table S2: Neurodegenerative changes in CA3 region by silver stain, Table S3: Neurodegenerative changes in CA1 region by FJC, Table S4: Neurodegenerative changes in CA3 region by FJC, Table S5A: Plasma level of MDA/4-HNE, Table S5B: Plasma level of Carbonyl: Table S6 Plasma detection of 3-Nitrotyrosine.

## Sample Availability

Samples of the compounds not available.

## References

[1] Block M.L., Zecca L., Hong J.S. (2007). Microglia-mediated neurotoxicity: Uncovering the molecular mechanisms. Nat. Rev. Neurosci..

[2] Migliore L., Coppedè F. (2009). Environmental-induced oxidative stress in neurodegenerative disorders and aging. Mutat. Res. Genet. Toxicol. Environ. Mutagenesis.

[3] Yang W., Omaye S.T. (2009). Air pollutants, oxidative stress and human health. Mutat. Res. /Genet. Toxicol. Environ. Mutagenesis.

[4] Calderón-Garcidueñas L., Leray E., Heydarpour P., Torres-Jardón R., Reis J. (2015). Air pollution, a rising environmental risk factor for cognition, neuroinflammation and neurodegeneration: The clinical impact on children and beyond. Rev. Neurol..

[5] Beckett W. (1991). Ozone, Air Pollution, and respiratory health. Yale J. Biol. Med..

[6] Bromberg P.A. (2016). Mechanisms of the acute effects of inhaled ozone in humans. Biochim. Biophys. Acta (BBA)-Gen. Subj..

[7] Miller D.B., Ghio A.J., Karoly E.D., Bell L.N., Snow S.J., Madden M.C., Soukup J., Cascio W.E., Gilmour M.I., Kodavanti U.P. (2016). Ozone Exposure Increases Circulating Stress Hormones and Lipid Metabolites in Humans. Am. J. Respir. Crit. Care Med..

[8] Huang T.T., Leu D., Zou Y. (2015). Oxidative stress and redox regulation on hippocampal-dependent cognitive functions. Arch. Biochem. Biophys..

[9] Rivas-Arancibia S., Zimbrón L.F., Rodríguez-Martínez E., Maldonado P.D., Borgonio Pérez G., Sepúlveda-Parada M. (2015). Oxidative stress-dependent changes in immune responses and cell death in the substantia nigra after ozone exposure in rat. Front. Aging Neurosci..

[10] Wang S., Irving G., Jiang L., Wang H., Li M., Wang X., Han W., Xu Y., Yang Y., Zeng T. (2017). Oxidative Stress Mediated Hippocampal Neuron Apoptosis Participated in Carbon Disulfide-Induced Rats Cognitive Dysfunction. Neurochem. Res..

[11] Barnham K.J., Masters C.L., Bush A.I. (2004). Neurodegenerative diseases and oxidative stress. Nat. Rev. Drug Discov..

[12] Roberts R.A., Laskin D.L., Smith C.V., Robertson F.M., Allen E.M., Doorn J.A., Slikker W. (2009). Nitrative and oxidative stress in toxicology and disease. Toxicol. Sci..

[13] Genc S., Zadeoglulari Z., Fuss S., Genc K. (2012). The Adverse Effects of Air Pollution on the nervous system. J. Toxicol..

[14] Martínez-Lazcano J.C., González-Guevara E., del Carmen Rubio M., Franco-Pérez J., Custodio V., Hernández-Cerón M., Livera C., Paz C. (2013). The effects of ozone exposure and associated injury mechanisms on the central nervous system. Rev. Neurosci..

[15] Calderón-Garcidueñas L., Franco-Lira M., Henríquez-Roldán C., Osnaya N., González-Maciel A., Reynoso-Robles R., Villarreal-Calderon R., Herritt L., Brooks D., Keefe S. (2010). Urban air pollution: Influences on olfactory function and pathology in exposed children and young adults. Exp. Toxicol. Pathol..

[16] Etsuo N. (2009). Lipid peroxidation: Physiological levels and dual biological effects. Free Radic. Biol. Med..

[17] Fedorova M., Bollineni C.R., Hoffmann R. (2013). Protein carbonylation as a major hallmark of oxidative damage: Update of analytical strategies. Redox Proteom..

[18] Poljsak B. (2011). Strategies for Reducing or Preventing the Generation of Oxidative Stress. Oxidative Med. Cell. Longev..

[19] Amalraj A., Pius A., Gopi S., Gopi S. (2016). Biological activities of curcuminoids, other biomolecules from turmeric and their derivatives - A review. J. Tradit. Complement. Med..

[20] Canales-Aguirre A.A., Gomez-Pinedo U.A., Luquin S., Ramírez-Herrera M.A., Mendoza-Magaña M.L., Feria-Velasco A. (2012). Curcumin protects against the oxidative damage induced by the pesticide parathion in the hippocampus of the rat brain. Nutr. Neurosci..

[21] De Alcântara G.F.T., Simoes-Neto E., da Cruz G.M., Nobre M.E., Neves K.R., de Andrade G.M., Brito G.A., Viana G.S. (2017). Curcumin reverses neurochemical, histological and immuno-histochemical alterations in the model of global brain ischemia. J. Tradit. Complement. Med..

[22] Li W., Suwanwela N.C., Patumraj S. (2016). Curcumin by down-regulating NF-kB and elevating Nrf2, reduces brain edema and neurological dysfunction after cerebral I/R. Microvasc. Res..

[23] Öz A., Çelik Ö. (2016). Curcumin inhibits oxidative stress-induced TRPM2 channel activation, calcium ion entry and apoptosis values in SH-SY5Y neuroblastoma cells: Involvement of transfection procedure. Mol. Membr. Biol..

[24] WHO (2010). Exposure to air pollution: A major public health concern. Preventing Disease through Healthy Environments.

[25] Rivas-Arancibia S., Guevara-Guzmán R., López-Vidal Y., Rodríguez-Martínez E., Zanardo-Gomes M., Angoa-Pérez M., Raisman-Vozari R. (2010). Oxidative Stress Caused by Ozone Exposure Induces Loss of Brain Repair in the Hippocampus of Adult Rats. Toxicol. Sci..

[26] Rivas-Arancibia S., Vazquez-Sandoval R., Gonzalez-Kladiano D., Schneider-Rivas S., Lechuga-Guerrero A. (1998). Effects of Ozone Exposure in Rats on Memory and Levels of Brain and Pulmonary Superoxide Dismutase. Environ. Res..

[27] Chen J.-C., Schwartz J. (2009). Neurobehavioral effects of ambient air pollution on cognitive performance in US adults. NeuroToxicology.

[28] Ye X., Carp R.I., Schmued L.C., Scallet A.C. (2001). Fluoro-Jade and silver methods: Application to the neuropathology of scrapie, a transmissible spongiform encephalopathy. Brain Res. Protoc..

[29] Switzer R.C. (2000). Application of Silver Degeneration Stains for Neurotoxicity Testing. Toxicol. Pathol..

[30] Connor A.J., Laskin J.D., Laskin D.L. (2012). Ozone-induced lung injury and sterile inflammation. Role of toll-like receptor 4. Exp. Mol. Pathol..

[31] Franklin J.L. (2011). Redox regulation of the intrinsic pathway in neuronal apoptosis. Antioxid. Redox Signal..

[32] Rodríguez-Martínez E., Nava-Ruiz C., Escamilla-Chimal E., Borgonio-Perez G., Rivas-Arancibia S. (2016). The Effect of Chronic Ozone Exposure on the Activation of Endoplasmic Reticulum Stress and Apoptosis in Rat Hippocampus. Front. Aging Neurosci..

[33] Block M.L., Calderón-Garcidueñas L. (2009). Air pollution: Mechanisms of neuroinflammation and CNS disease. Trends Neurosci..

[34] Chen X., Guo C., Kong J. (2012). Oxidative stress in neurodegenerative diseases. Neural Regen. Res..

[35] Radi E., Formichi P., Battisti C., Federico A. (2014). Apoptosis and Oxidative Stress in Neurodegenerative Diseases. J. Alzheimer’s Dis..

[36] Mukherjee A., Sarkar S., Jana S., Swarnakar S., Das N. (2019). Neuro-protective role of nanocapsulated curcumin against cerebral ischemia-reperfusion induced oxidative injury. Brain Res..

[37] Nery-Flores S.D., Mendoza-Magaña M.L., Ramírez-Herrera M.A., Ramírez-Vázquez J.J., Romero-Prado M.M.J., Cortez-Álvarez C.R., Ramírez-Mendoza A.A. (2018). Curcumin Exerted Neuroprotection against Ozone-Induced Oxidative Damage and Decreased NF-κB Activation in Rat Hippocampus and Serum Levels of Inflammatory Cytokines. Oxidative Med. Cell. Longev..

[38] Bucolo C., Drago F., Maisto R., Romano G.L., D’Agata V., Maugeri G., Giunta S. (2019). Curcumin prevents high glucose damage in retinal pigment epithelial cells through ERK1/2-mediated activation of the Nrf2/HO-1 pathway. J. Cell. Physiol..

[39] Sochocka M., Diniz B.S., Leszek J. (2017). Inflammatory Response in the CNS: Friend or Foe?. Mol. Neurobiol..

[40] Chen C., Arjomandi M., Balmes J., Tager I., Holland N. (2007). Effects of Chronic and Acute Ozone Exposure on Lipid Peroxidation and Antioxidant Capacity in Healthy Young Adults. Environ. Health Perspect..

[41] Jaroonwitchawan T., Chaicharoenaudomrung N., Namkaew J., Noisa P. (2017). Curcumin attenuates paraquat-induced cell death in human neuroblastoma cells through modulating oxidative stress and autophagy. Neurosci. Lett..

[42] Cross C.E., Motchnik P.A., Bruener B.A., Jones D.A., Kaur H., Ames B.N., Halliwell B. (1992). Oxidative damage to plasma constituents by ozone. FEBS Lett..

[43] Kodavanti P.R.S., Valdez M., Richards J.E., Agina-Obu D.I., Phillips P.M., Jarema K.A., Kodavanti U.P. (2020). Ozone-induced changes in oxidative stress parameters in brain regions of adult, middle-age, and senescent Brown Norway rats. Toxicol. Appl. Pharmacol..

[44] Borra S.K., Mahendra J., Gurumurthy P. (2014). Effect of Curcumin Against Oxidation of Biomolecules by Hydroxyl Radicals. J. Clin. Diagn. Res. JCDR.

[45] Wang Y.T., Piyankarage S.C., Williams D.L., Thatcher G.R. (2014). Proteomic Profiling of Nitrosative Stress: Protein S-Oxidation Accompanies S-Nitrosylation. ACS Chem. Biol..

[46] Sumanont Y., Murakami Y., Tohda M., Vajragupta O., Watanabe H., Matsumoto K. (2007). Effects of Manganese Complexes of Curcumin and Diacetylcurcumin on Kainic Acid-Induced Neurotoxic Responses in the Rat Hippocampus. Biol. Pharm. Bull..

[47] Lancaster J.R. (2006). Nitroxidative, Nitrosative, and Nitrative Stress:  Kinetic Predictions of Reactive Nitrogen Species Chemistry Under Biological Conditions. Chem. Res. Toxicol..

[48] Kumarathasan P., Blais E., Saravanamuthu A., Bielecki A., Mukherjee B., Bjarnason S., Guénette J., Goegan P., Vincent R. (2015). Nitrative stress, oxidative stress and plasma endothelin levels after inhalation of particulate matter and ozone. Part. Fibre Toxicol..

[49] Pinto-Almazán R., Rivas-Arancibia S., D Farfán-García E., Rodríguez-Martínez E., Guerra-Araiza C. (2014). Neuroprotective effects of tibolone against oxidative stress induced by ozone exposure. Rev. Neurol..

[50] Braidy N., Grant R., Adams S., Brew B.J., Guillemin G.J. (2009). Mechanism for Quinolinic Acid Cytotoxicity in Human Astrocytes and Neurons. Neurotox. Res..

[51] De Olmos J.S., Beltramino C.A., de Olmos de Lorenzo S. (1994). Use of an amino-cupric-silver technique for the detection of early and semiacute neuronal degeneration caused by neurotoxicants, hypoxia, and physical trauma. Neurotox. Teratol..

[52] Ahsan H. (2013). 3-Nitrotyrosine: A biomarker of nitrogen free radical species modified proteins in systemic autoimmunogenic conditions. Hum. Immunol..

[53] Ito T., Ogino K., Nagaoka K., Takemoto K. (2018). Relationship of particulate matter and ozone with 3-nitrotyrosine in the atmosphere. Environ. Pollut..

