# Supplementary material for: Curcumin Decreases Hippocampal Neurodegeneration and Nitro-Oxidative Damage to Plasma Proteins and Lipids Caused by Short-Term Exposure to Ozone
Source: Molecules. 2021 Jul 3;26(13):4075. doi: 10.3390/molecules26134075 (PMC8272084; doi:10.3390/molecules26134075)
Supplement: Supplementary file 1 [file molecules-26-04075-s001.zip › molecules-1238803-supplementary.pdf]

Supplementary material

Table S1. Neurodegenerative changes in CA1 region by silver stain

	Intact Control (%)	Curcumin Control (%)	Ozone Control (%)	Curcumin Preventive (%)
**1 hour**	0.000 ± 0.000	0.000 ± 0.000	1.778 ± 0.572	0.222 ± 0.147
**2 hours**	0.000 ± 0.000	0.000 ± 0.000	2.111 ± 0.351	0.778 ± 0.401
**4 hours**	0.111 ± 0.111	0.111 ± 0.111	3.667 ± 0.471	1.333 ± 0.408
**8 hours**	0.556 ± 0.242	0.444 ± 0.294	5.778 ± 0.662	2.222 ± 0.641

Table S2. Neurodegenerative changes in CA3 region by silver stain

	Intact Control (%)	Curcumin Control (%)	Ozone Control (%)	Curcumin Preventive (%)
**1 hour**	0.000 ± 0.000	0.000 ± 0.000	1.667 ± 0.373	1.333 ± 0.333
**2 hours**	0.222 ± 0.147	0.111 ± 0.111	1.778 ± 0.364	0.444 ± 0.242
**4 hours**	0.333 ± 0.167	0.333 ± 0.167	4.556 ± 0.801	1.889± 0.484
**8 hours**	0.444 ± 0.294	0.444 ± 0.242	7.667 ± 0.687	3.333 ± 0.289

Table S3. Neurodegenerative changes in CA1 region by FJC

	Intact Control (%)	Curcumin Control (%)	Ozone Control (%)	Curcumin Preventive (%)
**1 hour**	0.000 ± 0.000	0.111 ± 0.111	0.556 ± 0.242	0.111 ± 0.111
**2 hours**	0.222 ± 0.147	0.333 ± 0.167	1.222 ± 0.222	0.444 ± 0.176
**4 hours**	0.444 ± 0.176	0.222 ± 0.147	2.000 ± 0.373	0.778 ± 0.324
**8 hours**	0.556 ± 0.176	0.333 ± 0.167	2.556 ± 0.338	0.444 ± 0.176

Table S4. Neurodegenerative changes in CA3 region by FJC

	Intact Control (%)	Curcumin Control (%)	Ozone Control (%)	Curcumin Preventive (%)
**1 hour**	0.111 ± 0.111	0.111 ± 0.111	0.667 ± 0.236	0.111 ± 0.111
**2 hours**	0.222 ± 0.147	0.333 ± 0.167	1.111 ± 0.261	0.222 ± 0.147
**4 hours**	0.222 ± 0.147	0.555 ± 0.176	1.333 ± 0.287	0.556 ± 0.176
**8 hours**	0.670 ± 0.240	0.333 ± 0236	1.890 ± 0.350	0.440 ± 0.180

Table S5A. Plasma level of MDA/4-HNE

	Intact Control (nmol/mL)	Curcumin Control (nmol/mL)	Ozone Control (nmol/mL)	Curcumin Preventive (nmol/mL)
**1 hour**	2.491 ± 0.2704	2.203 ± 0.174	7.291 ± 0.526	3.537 ± 0.095
**2 hours**	1.604 ± 0.076	2.374 ± 0.124	8.037 ± 0.596	2.050 ± 0.152
**4 hours**	2.591 ± 0.058	2.233 ± 0.147	8.630 ± 0.498	2.849 ± 0.020
**8 hours**	3.684 ± 0.045	1.281 ± 0.124	10.7660 ± 0.522	2.990 ± 0.200

Table S5B. Plasma level of Carbonyl

	Intact Control (nmol/mg)	Curcumin Control (nmol/mg)	Ozone Control (nmol/mg)	Curcumin Preventive (nmol/mg)
**1 hour**	0.249 ± 0.011	0.277 ± 0.006	0.519 ± 0.016	0.437 ± 0.004
**2 hours**	0.314 ± 0.016	0.312 ± 0.008	0.653 ± 0.014	0.519 ± 0.007
**4 hours**	0.302 ± 0.014	0.324 ± 0.008	0.789 ± 0.006	0.538 ± 0.002
**8 hours**	0.377 ± 0.006	0.305 ± 0.017	0.993 ± 0.009	0.610 ± 0.008

Table S6 Plasma detection of 3-Nitrotyrosine

	Intact Control (IOD)	Curcumin Control (IOD)	Ozone Control (IOD)	Curcumin Preventive (IOD)
**1 hour**	756800 ± 114624	413800 ± 133372	4536000 ± 814242	3228000 ± 143997
**2 hours**	653000 ± 197026	629000 ± 80115	4896000 ± 392619	4682000 ± 243195
**4 hours**	1002000 ± 500746	1469000 ± 174584	7866000 ± 976533	5118000 ± 86059
**8 hours**	2382000 ± 282492	2419000 ± 413580	8024000 ± 146500	5188000 ± 247147
